# Dietary Arsenic Exposure: Xue et al. Respond

**DOI:** 10.1289/ehp.1002328R

**Published:** 2010-08

**Authors:** Jianping Xue, Valerie Zartarian, Shi V. Liu, Sheng-Wei Wang, Panos Georgopoulos

**Affiliations:** U.S. Environmental Protection Agency, Office of Research and Development, National Exposure Research Laboratory Research Triangle Park, North Carolina, E-mail: xue.jianping@epa.gov; Graduate Institute of Environmental Health, National Taiwan University, Taipei, Taiwan; Environmental and Occupational Health Sciences Institute, Piscataway, New Jersey

In our article (Xue et al. 2010), we cited Petito Boyce et al. (2008) based on their major conclusion stated at the end of their abstract that, “typical and high-end background exposures to inorganic arsenic in U.S. populations do not present elevated risks of carcinogenicity.” We agree with Petito Boyce et al. that we “missed an opportunity to provide additional support for” our overall conclusions, and very much appreciate that they have offered this detailed comparison showing the agreement between our modeling results.

Our discussion of Petito Boyce et al. (2008)’s conclusions was intended to bolster the need to develop a more comprehensive analysis of the sources of inorganic arsenic exposure, not to suggest that their exposure analysis was incomplete or inaccurate.
